# Benefits of A2 Milk for Sports Nutrition, Health and Performance

**DOI:** 10.3389/fnut.2022.935344

**Published:** 2022-07-13

**Authors:** Merve Kaplan, Barış Baydemir, Bilgetekin Burak Günar, Ayşenur Arslan, Hatice Duman, Sercan Karav

**Affiliations:** ^1^Department of Molecular Biology and Genetics, Canakkale Onsekiz Mart University, Canakkale, Turkey; ^2^Department of Coaching Education, Canakkale Onsekiz Mart University, Canakkale, Turkey; ^3^Department of Physical Education and Sports Teaching, Canakkale Onsekiz Mart University, Canakkale, Turkey

**Keywords:** bovine milk, A2 milk, A2 β-casein, sports nutrition, health, athlete performance

## Abstract

Bovine milk is one of the best pre-and pro-workout sources for athletes owing to its rich nutritional content. Even though bovine milk consumption significantly benefits athletes' health and performance, many athletes cannot consume bovine milk since they struggle with gastrointestinal problems caused after milk consumption. Especially, the consumption of regular milk, which contains A1 β-casein, is associated with a variety of diseases ranging from gastrointestinal discomfort to ischemic heart diseases. The main reason behind this is related to β-casomorphine 7 (BCM-7), which is derived from A1 β-casein during the digestion of A1 milk. A1 β-casein is formed as a result of a point mutation in the position of 67^th^ in the amino acid sequence A2 β-casein by changing proline to histidine. Therefore, this mutated form of β-casein in regular milk cannot easily be digested by the human-associated digestion enzymes. A2 milk, which includes A2 β-casein instead of A1 β-casein, is the best substitute for regular milk with the same nutritional content. This natural form of milk positively affects the athlete's health as well as performance without causing any gastrointestinal discomfort or more serious problems which are seen in the consumption of regular milk. In this review, A2 milk and its potential health effects in comparison to diseases related to A1 milk consumption are discussed.

## Introduction

Bovine milk and its products have been considered a remarkable nutritional source that improves human health in a variety of ways (1–6). Bovine milk has become an ideal recovery drink among athletes since it is a unique source of proteins, lipids, amino acids, and minerals that are not included in other sports beverages (7–11). Many studies have clearly shown that bovine milk has a critical impact on athlete health in several aspects owing to its rich nutritional content (12–16). The consumption of bovine milk positively affects endurance, muscle strength, muscle damage, and recovery in athletes. Furthermore, bovine milk consumption after physical exercise has a positive effect on acute recovery, as well as chronic exercise adaptation (12, 13, 17, 18).

The consumption of suitable foods is critical for athletes to meet the optimal energy needs during intense loads in branch-specific training and to ensure rapid recovery after exercise (19). Thus, many athletes prefer to consume bovine milk and its products which provide a myriad of health benefits through its rich nutritional content (8). From a nutritional perspective, bovine milk contains carbohydrates (lactose) in a similar amount to carbohydrate levels in many sports drinks, which provides the necessary energy to the athletes during the exercise (20, 21). It also includes bioactive proteins such as whey and casein (3:1 ratio) that enhance the level of amino acid in the serum, muscle protein synthesis rate, as well as muscle damage repair (22). In addition, bovine milk is an alternative hydration drink to other sports drinks due to its high concentration of some minerals namely sodium and potassium, which helps the skeletal muscle recovery (23, 24).

**Figure 1:**
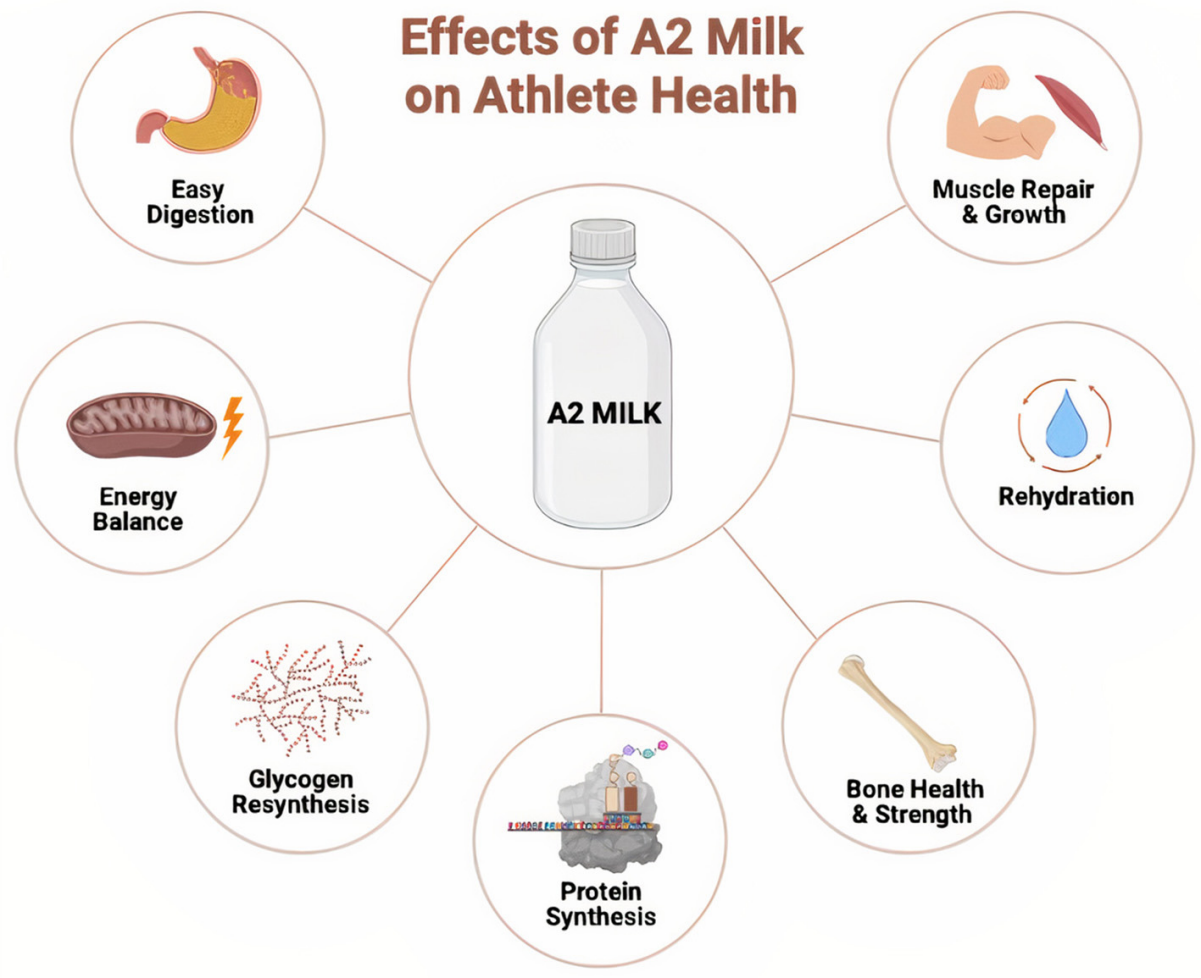
Different possible effects of the consumption of A2 milk on athlete health.

On the other hand, a significant number of athletes cannot easily digest regular bovine milk and therefore, exclude it from their diet. The digestion of regular bovine milk, which includes A1 β-casein, can cause lactose intolerance and potential health problems mainly linked to its A1 β-casein and its derived peptide β-casomorphine 7 (BCM-7). Moreover, this small peptide formed during the digestion of A1 milk has been linked with several health problems from intolerance to diabetes (25). Thus, regular A1 milk and based products have been excluded from the sports diet even though they benefit athlete health in several ways. The lack of bovine milk in the diet of athletes means that they miss out on essential nutrients including protein, vitamin D, calcium, and potassium for their performance (12, 20). An alternative for athletes who have GI discomfort or other health problems due to the consumption of regular milk is a critical requirement. Therefore, A2 milk, which lacks A1 β-casein and related BCM-7 expression, is considered to be a promising alternative to regular A1 milk for athletes who cannot consume it (26–28). A2 bovine milk not only offers all the health benefits of regular milk but also provides easy digestion for athletes (Figure 1). The main scope of this review is to summarize the potential benefits of A2 milk on human health as an alternative sport drink to regular milk. In addition, the review aims compherensively discuss the relationship between regular milk consumption by atheletes and various diseases such as gastrointestinal discomfort, cardiovascular diseases, and type 1 diabetes.

## A2 Milk–an Alternative Bovine Milk for Athletes

The easy-digest A2 milk is a noticeable alternative for athletes who come to face health problems including GI discomfort when they consume A1 milk (20–22). A2 milk does not cause such serious health problems; besides, it has nearly the same nutritional composition as regular milk (29–32). Regarding bovine milk composition, it includes approximately 87% water and 13% such milk solids as lactose, fat, proteins, and minerals. Milk proteins consist of 20% whey (a-lactalbumin, β -lactoglobulin, transferrin, albumin, and lactoferrin) and 80% casein proteins (α, β, κ- casein) (33, 34). Caseins, which exert significant biological roles, include 45% β-casein has great amino acid balance. β-casein concentrations in A1 and A2 milk are 8.59 and 8.02 mg/mL, respectively. β-casein includes 209 amino acid residues of which 16.7% are proline which causes a limitation in the formation of an α-helix. Therefore, a variety of mutations resulted in different variants of β-casein including A1, A2, A3, B, C, D, E, F, G, H1, H2, and I (35).

A1 and A2 forms are the most common β-casein variants among other β-casein forms (35). They include almost the same composition in terms of protein, fat, carbohydrate, and other contents concentration (Table 1). The only difference between A1 and A2 variants stems from a point mutation which causes the difference in a single nucleotide at the 67^th^ position of the β-casein gene. A2 β-casein includes proline at the 67^th^ position of its amino acid sequence. However, this amino acid was converted to histidine by a mutation which causes the formation of the A1 β-casein variant (23). Bovines, therefore, produce A1 or A2 milk depending on the β-casein form they have (37).

**Table 1:** Comparison of the nutritional content of A1 and A2.

**Component**	**A2 Milk**	**A1 Milk**	**References**
Energy (kJ/100 mL)	278	270	(25)
Protein (mg/mL)	~33	~33	(25)
α_s_-casein	16.37	16.08	(36)
β-casein	8.02	8.59	(36)
κ-casein	2.44	2.41	(36)
β-lactoglobulin	4.50	4.49	(36)
α-lactalbumin	1.46	1.43	(36)
Serum albumin	0.45	0.46	(36)
Immunoglobulins	0.47	0.48	(36)
Fat (mg/mL)	37	35	(25)
Carbohydrate (mg/mL)	50	48	(25)
Sodium (mg/mL)	0.37	0.45	(25)
Calcium (mg/mL)	1.17	1.20	(25)

Several studies have shown that GI discomfort and other health problems, which are also serious problems many athletes experience when they consume A1 milk, are closely related to BCM-7 peptide (38–40). However, as A2 milk lacks A1 β-casein and derived BCM-7, does not cause such health concerns for athletes. While the digestion of A1 protein causes the formation of BCM-7; (Tyr-Pro-Phe-Pro-Gly-Pro-Ile), A2 protein digestion forms another peptide called β-casomorphin-9 (BCM-9; Tyr-Pro-Phe-Pro-Gly-Pro-Ile-Pro-Asn) (23). The proline at the 67^th^ position of A2 β-casein hinders the BCM-7 formation in the human body, but histidine in A1 β-casein at the same position allows the cleavage by gastrointestinal enzymes to form BCM-7. This morphine-like small peptide cannot be digested by human-associated enzymes, which causes indigestion problems. BCM-7 molecule was not observed in the urine or blood samples of A2 cows since A2 β-casein is broken down into peptides and then amino acids by an easy digestion process (27, 41). In A1 milk consumption, in contrast, the peptides cannot be broken down into amino acids, which causes the transition of this molecule into the gastrointestinal tract and bloodstream through leakages in the gut (25). Several studies including epidemiological and clinical research have supported that the BCM-7 is a risk for diseases such as gastrointestinal discomfort, type 1 diabetes, ischemic heart, and, neurological diseases (42–46). Consequently, athletes may suffer not only from GI discomfort but also from health problems caused by A1 milk consumption. As an alternative drink for athletes, A2 milk is a promising option to be benefited from the nutritional content of bovine milk without health problems.

## Impact Of A1 And A2 Milk on Athlete Performance And Health

Milk and dairy products are associated with the concepts of physical activity, exercise, training, and health (2). The use of milk rich in amino acids, which is necessary for energy, shows branch-depending differences in athletes. Although its health benefits are known, the thought that it will negatively affect performance drives athletes away from milk. The number of studies on this subject has started to increase day by day. It is seen that milk taken after high-intensity interval running protocol application in endurance athletes does not negatively affect the nutrition protocol in the recovery process in athletes (47). Athletes have to train continuously to maximize their performance. According to the competition calendar, athletes who train twice a day in some periods physiologically need a fast recovery period. Studies are showing that milk has positive effects on athlete performance as well as other supplements that accelerate recovery (48). However, athletes with intolerance to dairy products are subject to a diet devoid of milk and dairy products in the cycle of regaining the lost energy after training. However, it is emphasized that A2 milk will be a suitable alternative since it does not contain the protein in A1 milk (20).

It is unclear whether A2 milk has a different effect on performance elements such as strength, speed, and endurance in comparison to regular milk. However, A2 milk might have a similar positive effect on athlete performance as regular milk because they have the same nutritional value. In addition to the positive effect of A2 milk on athlete performance quality, it can be a precise alternative for athletes who have some medical issues such as gastrointestinal discomfort, diabetes, and cardiovascular and neurological diseases.

## Exercise Performance

As for the impact of A2 milk on athlete health, several studies are focused on muscle damage and recovery which are crucial for the quality of exercise performance. In a research study, Kirk et al. (49) compared the effects of A1 and A2 milk on 20 m sprint, vertical jump, and exercise-induced muscle damage. According to the study results, both milk forms had a similar effect on sprint, vertical jump performance, and post-exercise recovery, which means that A2 milk may be a good substitute for lactose intolerant athletes (20).

In another study, regular chocolate milk and A2 milk had similar effects on recovery after physical exercise (50). In addition, A2 milk consists of more proline amino acids due to the difference at the 67^th^ amino acid in the casein structure between A1 and A2 milk (51, 52). Proline is a multifunctional amino acid classified as one of the non-essential amino acids. It is one of the gluconeogenic amino acids and may increase endurance performance by protecting blood sugar and hepatic glycogen levels, especially in long-term endurance exercises (53). Furthermore, proline is a critical amino acid for protein synthesis and cell growth. It also plays an important role in osmoregulation, redox signaling, protein stability, cellular bioenergetics, and stress resistance (54). In addition to having all the nutritional values of A1 milk, A2 milk can be preferred by all athletes due to its easy-to-digest property and higher proline content.

## Gastrointestinal Discomfort

Gastrointestinal disorders are common amongst athletes with a rate of up to 70% (55, 56). Most of the athletic population including runners, weightlifters, cyclists, and triathletes experience the discomfort problem due to mainly upper GI complaints such as nausea, vomiting, heartburn, and epigastric pain. Nutrition is of utmost importance to tackle these GI problems; however, inaccurate nutrition deteriorates symptoms. A1 milk, for instance, can cause symptoms associated with milk intolerance such as stool frequency, fecal and serum biomarkers, constipation, and transit time (32, 35, 37, 38).

Several studies have shown that A1 milk causes some gastrointestinal discomfort problems during its digestion. A study has indicated that A1 milk causes higher stool consistency according to Bristol Scale in comparison to A2 milk. The study also showed that abdominal pain and stool consistency is positively associated with A1 milk consumption (r = 0.52), but this is not observed for A2 milk digestion (r = −0.13) (57). A study also showed that A2 milk consumption by lactose-intolerant individuals significantly diminished the intolerance symptoms (23). In another study, lactose intolerant individuals consumed A2 milk, A1 milk, regular milk without lactose, and Jersey milk to evaluate their gastrointestinal symptoms and hydrogen production during digestion. They showed that A2 milk causes considerably fewer gastrointestinal symptoms and pain (27). Another study also examined lactose intolerance symptoms after participants consume A1/A2 milk and only A2 milk. The study is resulted by that the group which consumes A2 milk presents fewer intolerance symptoms. The consumption of A1/A2 milk was linked with post-dairy digestive discomfort and a high proportion of inflammation markers and BCM-7 (35). A study performed on animals similarly showed that BCM-7 exerts different impacts on gastrointestinal function such as declining the frequency and amplitude of intestinal contractions. Barnett et al. (58) showed that A1 milk feeding on rats has an increment on myeloperoxidase which is an inflammatory marker with 65%. Generally, it is mainly shown that A1 milk consumption causes systemic inflammation and gastrointestinal mobility related to BCM-7 formation during its digestion (58).

In contrast, A2 milk consumption was not correlated with post-dairy discomfort, and it is considered to milk can be easily consumed without any gastrointestinal discomfort (20). A2 milk consumption by 10 individuals who are not tolerant to A1 milk did not cause any gastrointestinal problems. Another study also showed that A2 milk diminished gastrointestinal-related symptoms of lactose intolerant, whereas A1 milk decreased lactase activity and enhanced symptoms (25). This easy digest product is also highly preferred by athletes to consume as an energy source after their exercise. In a study related to A2 milk and athlete health, the effect of A2, regular milk, and placebo on exercise-induced muscle damage is evaluated in a group including 21 men who regularly run. The results showed that A2 milk consumption diminishes muscle function loss and improves the recovery period (49). Thus, alternate A2 milk may be a favorable drink for athletes without causing any GI concerns.

## Type 1 Diabetes

Type 1 diabetes, which is a form of diabetes mellitus, is caused by a lack of insulin due to the problem in β-cells producing insulin in the islets of Langerhans of the pancreas (59). Many athletes with type 1 diabetes are at risk of hypoglycemia during and post-exercise (60, 61). Therefore, this risky situation may hinder the sports career of athletes with type 1 diabetes. Another considerable point is that the consumption of popular post-exercise drink A1 milk can worsen symptoms related to type 1 diabetes. As regards studies on Type 1 diabetes and A1 β-casein, A1 milk causes worse symptoms of Type 1 diabetes due to BCM-7 formation, whereas it is not observed in A2 milk consumption does not cause morphine-like peptide BCM-7 release (26, 41).

The relationship between A1 milk consumption and type 1 diabetes has been debated for long years (26, 42, 43, 62). Animal studies have shown that no difference between A1 and A2 milk consumption caused to high risk of type 1 diabetes. However, A1 milk intake by susceptible rats increases the risk of type 1 diabetes. Regarding studies on humans, a specific human leukocyte antigen (HLA-DR) may be at high risk of developing Type 1 diabetes due to cow's milk consumption (63). Another study represented that the incidence of type 1 diabetes was not strongly correlated with total protein consumption (r = +0.402), whereas A1 milk consumption was (r = +0.726) (64). Furthermore, Laugesen and Elliott showed a positive association (r = 0.92) between A1 milk supply by cows per capita and type 1 diabetes in 19 countries. This noticeably indicated that Finland and Sweden's highest A1 milk intake per capita had a higher incidence rate, while low frequencies were in Venezuela and Japan where the lowest A1 milk consumption takes place per capita (44).

## Cardiovascular Diseases

Cardiovascular diseases, to date, have been one of the major causes of mortality and morbidity at the global level. Although precise nutrition and physical exercise are recommendations for the prevention of such diseases, even elite athletes are prone to developing cardiovascular diseases as they age (65, 66). Some drinks in athletes' diets pre- or pro- exercise were correlated with the symptoms of cardiovascular diseases. Especially, A1 milk was associated with some markers of cardiovascular diseases such as atherosclerosis, plasma cholesterol level, and oxidation of low-density lipoprotein (43, 67–69).

Several studies have shown that A1 milk consumption was correlated with ischemic heart disease mortality in France, Northern Ireland, and West Germany (r^2^ = 0.86) (43, 44). Similarly, A1 β-casein per capita by milk and cream was strongly associated with ischemic heart disease in 20 different countries. BCM-7 formed after A1 milk consumption is also linked with the oxidation process of low-density lipoprotein (LDL). Macrophages absorb oxidized LDL molecules with surface receptors and converted them into foam cells, which promotes atherosclerosis in the heart (70). Another study also showed that A1 milk is more responsible for the atherosclerosis process in comparison to A2 milk. An artificial injury, in animal models, was made in the carotid artery of rabbits and was fed on A1 and A2 milk. The result of the study showed that rabbits fed on A1 milk had thicker streaks with fatty structures on the injured area than rabbits fed on A2 milk (71). Consequently, A2 milk can be preferred for athletes who have or are at risk of cardiovascular diseases instead of regular A1 milk.

## Neurological Diseases

Today, many people including many athletes suffer from various neurological diseases which seriously affect their quality of life. Balanced nutrition is a major factor that strongly affects symptoms of some neurological diseases, which could be a good way to be neurologically healthy. In contrast, BCM-7 derived from the consumption of A1 milk was correlated with the symptoms of some neurological diseases (40, 72, 73).

Studies related to A1 β-casein-derived BCM-7 show a relationship between this small peptide and a variety of neurological problems. In a study, for instance, BCM-7 in different concentrations is injected into 35 rats to examine its effects on rats' brain functions. It showed that BCM-7 can pass the blood-brain barrier, and even more, it can activate brain cells which causes some anatomic and functional changes in brain cells (72). The relationship between BCM-7 and genes related to atopic dermatitis is also examined in another study. In this study, the MOR gene responsible for an opioid receptor which is associated with the negative effect of BCM-7 on digestion, immunity, and the nervous system was found as significantly more active because of the A1 milk consumption. In addition, the activity of the DPP4 gene, which is responsible for the production of a protein that degrades BCM-7, is decreased in dermatitis patients. In milk variants, furthermore, the highest BCM-7 concentration is observed after the hydrolysis of A1 milk (51). Furthermore, autism, which is an autism spectrum disorder, is characterized by social and behavioral problems. Some studies have shown that BCM-7 may worsen the symptoms of autism development. The worse situations of neurological symptoms have been associated with the consumption of A1 milk and wheat by autistic patients (73, 74). It can clearly be stated that there is a critical need for further investigations on A1 milk consumption and neurological diseases including autism.

## A2 Milk-Based Meals for Athletes

Many athletes have been excluded A1 milk and products from their diet because of a variety of health problems ranging from intolerance to diabetes (52, 64, 69, 71, 75). Therefore, they are more prone to consuming other non-dairy alternatives such as almond or oat milk; however, these plant-based options do not include the same nutritional content and health benefits as bovine milk. Many lactose-intolerant athletes do not experience gastrointestinal problems when they ingest plant-based milk and products. However, they lack a variety of benefits of regular dairy due to its rich nutritional content (76).

Nowadays, A2 milk and products have been introduced to the diet plan of many athletes to benefit them in many aspects of health with almost the same nutritional content as regular milk. Due to the easy-digest property of A2 milk, it is a source to make A2 milk-based meals for athletes pre- and pro-workout. Moreover, several popular dieticians have started to recommend the integration of A2 milk into athletes' diets in many ways (27, 77). For instance, A2 milk can be used to prepare a post-workout snack by mixing a piece of fruits or a pre-workout snack having it with cereal or muesli. Eventually, A2 milk-based meals may offer a precise alternative to before and after exercise food sources with important health benefits.

## Conclusion

Milk has an important part of the sports diet thanks to its rich nutritional elements. However, many athletes cannot consume milk due to GI discomfort after digestion. A2 milk is a considerable alternative for athletes with such ailments. A2 dairy allows athletes to take the nutrients they can get from regular milk without any discomfort. In this review, the effects of A2 milk, which has become more important in recent years, on athletes are compressively discussed. A2 milk has noticeable positive effects on both athlete health and performance. Consumption of A2 milk has a lower risk compared to A1 milk against digestive problems, type 1 diabetes, cardiovascular diseases, and neurological diseases, which have an important place in terms of the general health status of athletes. Its similarity with regular milk in athlete performance makes A2 milk a reliable food for athletes with GI disorders. A2 milk has a higher potential to be used as a nutritional source in athletes and even more in all humans' diets owing to its incredible functions on health. A2 milk and its derived products ranging from cheese to yogurt would be an important part of a healthy and balanced diet of a significant number of people in the future. This review paper, therefore, is of utmost importance to better understand the positive impact of A2 milk on athlete health. However, decomposing effects of regular milk and A2 milk on different physical performances such as strength, speed, and endurance are not fully known. Therefore, there is a critical need for more *in-vitro* and *in-vivo* studies comparing the effects of regular milk and A2 milk on sportive performance.

## Author Contributions

SK organized the general content of the paper. MK was responsible for general editing and organizing the authors as well as the contribution for three sections. BB and BG were responsible for one section of the paper. AA and HD contributed one section of the paper. All authors contributed to the article and approved the submitted version.

## Funding

Uluova Süt Ticaret A.S. (Uluova Milk Trading Co.) has funded this study.

## Conflict of Interest

SK has received funding from Uluova Süt Ticaret A.S. (Uluova Milk Trading Co.). The remaining authors declare that the research was conducted in the absence of any commercial or financial relationships that could be construed as a potential conflict of interest.

## Publisher's Note

All claims expressed in this article are solely those of the authors and do not necessarily represent those of their affiliated organizations, or those of the publisher, the editors and the reviewers. Any product that may be evaluated in this article, or claim that may be made by its manufacturer, is not guaranteed or endorsed by the publisher.
